# Visual word form processing deficits driven by severity of reading impairments in children with developmental dyslexia

**DOI:** 10.1038/s41598-020-75111-8

**Published:** 2020-10-30

**Authors:** S. Brem, U. Maurer, M. Kronbichler, M. Schurz, F. Richlan, V. Blau, J. Reithler, S. van der Mark, E. Schulz, K. Bucher, K. Moll, K. Landerl, E. Martin, R. Goebel, G. Schulte-Körne, L. Blomert, H. Wimmer, D. Brandeis

**Affiliations:** 1 Department of Child and Adolescent Psychiatry and Psychotherapy, Psychiatric Hospital, University of Zurich, Neumuensterallee 9, 8032 Zurich, Switzerland; 2 Neuroscience Center Zurich, University of Zurich and ETH Zurich, Zurich, Switzerland; 3 Department of Psychology, The Chinese University of Hong Kong, Hong Kong, China; 4 Brain and Mind Institute, The Chinese University of Hong Kong, Hong Kong, China; 5 Centre for Cognitive Neuroscience and Department of Psychology, University of Salzburg, Salzburg, Austria; 6 Neuroscience Institute, Christian Doppler Clinic, Paracelsus Medical University, Salzburg, Austria; 7 Cognitive Neuroscience Department, Faculty of Psychology and Neuroscience, Maastricht University, Maastricht, The Netherlands; 8 Maastricht Brain Imaging Center (M-BIC), Faculty of Psychology and Neuroscience, Maastricht University, Maastricht, The Netherlands; 9 MR-Center, University Children’s Hospital, University of Zürich, Zurich, Switzerland; 10 Department of Neurology, Ludwig-Maximilians-Universität München, Munich, Germany; 11 Department of Child and Adolescent Psychiatry, Psychosomatics, and Psychotherapy, University Hospital, Ludwig-Maximilians-University Munich, Munich, Germany; 12 Department of Psychology, University of Salzburg, Salzburg, Austria; 13 Institute of Psychology, University of Graz, Graz, Austria; 14 Department of Child and Adolescent Psychiatry and Psychotherapy, Central Institute of Mental Health, Medical Faculty Mannheim, Heidelberg University, Mannheim, Germany

**Keywords:** Cognitive neuroscience, Reading

## Abstract

The visual word form area (VWFA) in the left ventral occipito-temporal (vOT) cortex is key to fluent reading in children and adults. Diminished VWFA activation during print processing tasks is a common finding in subjects with severe reading problems. Here, we report fMRI data from a multicentre study with 140 children in primary school (7.9–12.2 years; 55 children with dyslexia, 73 typical readers, 12 intermediate readers). All performed a semantic task on visually presented words and a matched control task on symbol strings. With this large group of children, including the entire spectrum from severely impaired to highly fluent readers, we aimed to clarify the association of reading fluency and left vOT activation during visual word processing. The results of this study confirm reduced word-sensitive activation within the left vOT in children with dyslexia. Interestingly, the association of reading skills and left vOT activation was especially strong and spatially extended in children with dyslexia. Thus, deficits in basic visual word form processing increase with the severity of reading disability but seem only weakly associated with fluency within the typical reading range suggesting a linear dependence of reading scores with VFWA activation only in the poorest readers.

## Introduction

Reading words correctly and fluently is a prerequisite for understanding written text. Like other scholastic skills, reading shows a normal distribution within the population^1^. The poorest 5–10% of the children with insufficient mastery of reading are considered as presenting with developmental dyslexia^2^. While phonological deficits have been suggested to play a central role for the impairments seen in dyslexia^3^, processing visual words for meaning remains the central task in reading. Thus, understanding how poor readers and especially children with dyslexia process visual words in the context of a semantic task is essential, and functional neuroimaging studies offer insight into the neural mechanisms involved.

It is undisputed that the left ventral occipito-temporal cortex (vOT) has a prominent role in processing print^4–15^ and consequently in reading for meaning. Especially an area within the left mid-fusiform gyrus, often referred to as the visual word form area (VWFA) typically shows a preference in responding to words or orthographic stimuli as compared to visual control stimuli such as checkerboard patterns^7^ even though this region is also involved in other processes^16,17^. Subsequent studies extended the notion of a VWFA to an entire visual word form system (VWFS) in the inferior occipito-temporal cortex that is progressively tuned to orthographic regularities with more anterior locations^14,18–22^ and a slightly more posterior region to encode letters (letter form area: LFA^23^). Even more fine grained functional divisions of the complex left vOT system along the posterior-anterior and lateral-medial dimensions were detected in more recent studies: Functional and microarchitectonic properties divide the larger visual word form system into a posterior perceptual word form area responsible for feature extraction and a more anterior lexical integration site corresponding approximately to the location of the classic VWFA^24^. Lately, Bouhali and colleagues suggested an additional lateral to medial functional segregation of the vOT, whereby multiletter sublexical graphemes are processed in medial regions in comparison to the lexico-semantic encoding in more lateral sites^25^.

Developmental studies indicate that the print-sensitive response of the VWFA shows a rapid development in the first few months and years of learning to read^26–33^ and that it is initiated by grapheme-phoneme correspondence training in children^26,29,34^ and adults^35,36^. The location of the emerging print-sensitive area within the vOT seems constrained by its established connectivity to language regions long before children learn to read at school^13,37^ and develops in cortical patches with only weak specialization^29^. The exact location of the VWFA within the left vOT is subject to individual variations^10,29,33,38^. Importantly, the strength of the print sensitive response in the VWFA is modulated by the expertise level of reading in children and adults^9,27,28,30,31,33^. It is for this reason that the VWFA has also been referred to as reading skill zone^39,40^. Meta-analyses on children and adults with reading impairments revealed some of the most robust and consistent functional alterations in left vOT areas when tasks involve reading. Poor readers such as those with dyslexia activate this region less than typical readers^41–43^. This deficient activation in poor readers seems convergent for shallow and deep alphabetic languages^44,45^ and even generalizes to different writings systems^46^. Disruption of the activation in the VWFS through stimulation or through acquired brain lesions^47^ results in impaired reading^48^, again emphasizing its critical role for fluent reading.

Taken together, recent evidence points to a pivotal role of the VWFA for reading and reading acquisition^49^. The visual word processing system in the left vOT is sensitive to the perceptual and lexical properties of words^23–25,31^ and sublexical units^23,25^. This system is, however at the same time strongly influenced by top-down input from higher level phonological and semantic language areas^19,50^ and the dorsal attention network^51^. Therefore, also task demands modulate the differences seen between typical readers and subjects with dyslexia in the left vOT. Several studies reporting reduced left vOT activation in dyslexia for example employed tasks that put strong demands on phonological processing, such as reading pseudowords^45,52–55^, reading pseudohomophones^22,54^, or reading words having orthographic-phonological conflicts^56^. Studies employing explicit or implicit reading tasks with regular words typically reported less robust underactivation in left vOT in dyslexia (^57–60^; but see^61^), although deviant left vOT activation could still be detected in more focal ROI analyses^22,58–60^ or when comparing basic word processing and not print-sensitivity contrasts among groups^30^. While these studies emphasize that reading impairments are strongly associated with hypoactivation in inferior occipito-temporal regions under certain task conditions, it is less clear to what extent such deviations occur in more natural reading situations when readers focus on the meaning of words.

Some ambiguity in the dyslexia results might also stem from the typically small samples and the often-used categorical approaches in neuroimaging studies. Large-scale imaging studies on reading are still rare, and so far often examined tasks challenging the phonological system^62–64^. Thus, the goal of the current study was to investigate word reading in a large sample of children at varying reading skills in a primarily dimensional approach using a task that emphasizes semantic processing, i.e. reading for meaning. The use of our large sample allowed us to use reading fluency as a continuous regressor in the analyses instead of examining just categorical differences.

In order to obtain a large sample of subjects we developed a short controlled reading task in a multi-center study (NEURODYS). This task not only included visual words in the context of a semantic task, but also a low-level visual control task (cross-hatches and stars), where subjects had to decide whether all characters were the same or not [similar to^52,58,63,65^]. Data analyses focussed on the modulation of print-sensitivity through reading skills across the entire multi-center sample while controlling for differences between sites.

## Methods

### Subjects

From an initial group of 182 children with functional (fMRI) and structural (sMRI) data collected in three sites across Europe, 42 children were excluded, because of poor fMRI data quality or excessive movement (n = 18), because of prominent attention-deficit/hyperactivity disorder (ADHD) symptoms (n = 10), low IQ (n = 6) or absence of behavioural log files (n = 8). The analyses for the remaining 140 children (age 7.9–12.2 years) who participated either in Zurich (ZRH, n = 80), Maastricht (MAS, n = 35), or Salzburg (SBG, n = 25) are reported in this paper.

Children whose reading fluency score (correct words per minute) was more than 1.25 SD below the normative mean were considered dyslexic (< 10.5 percentile, n = 55). Children with reading fluency scores ≥ − 0.85 SD below the normative mean were considered typical readers (≥ 19.7 percentile, n = 73). Children (n =   12)  having a  z-score  between − 1.25 and − 0.85 formed the gap group of “intermediate readers”. These intermediate readers were included for correlational analyses but excluded for categorical group comparisons. All children had an estimated nonverbal and verbal IQ of at least 85 based on the block design and similarities subtests of the WISC^66^. No child was excluded due to poor task performance if behavioural data were available.

Reading skills were assessed with the “Salzburger Lese- und Rechtschreibtest II” (SLRT-II^67^) in Zurich and Salzburg, and with the Dyslexia Differential Diagnostics^68^ in Maastricht.

Prominent ADHD symptoms were either a clinical diagnosis (reported by the parents), or T-scores ≥ 66.5 in the German version of the Child Behaviour Checklist (CBCL) attention scale (^69^ only available for Zurich and Salzburg).

For covariance analyses that used reading as a covariate instead of categorical groups the z-score (relative to the norms) of the word fluency measure was used.

As indicated in Table 1, the group of children with dyslexia differed from the group of typical readers in reading fluency, but not in IQ, age, sex, or handedness.

**Table 1:** Demographic data, behavioural data on reading competence and fMRI task performance.

Variables	Typical (TYP)	Dyslexia (DYS)	Intermediate	*t-*test TYP-DYS
	M (SD)	M (SD)	M (SD)	p
**Behavioural and demographic measures**				
Site ZRH:SBG:MAS (n)	45:13:15	27:11:17	8:1:3	
Sex (male:female) (n)	37:36	30:25	8:4	0.722^b^
Handedness (r:l) (n)	59:14	48:7	9:3	0.470^b^
Age (years)	10.05 (1.14)	9.87 (1.33)	10.2(1.6)	0.372
School grade	4.1 (1.4)	4.2 (1.3)	4.4 (1.4)	0.444
Word reading (SLRT-II) fluency^a^	0.4 (0.93)	− 1.8 (0.44)	− 0.99 (0.13)	** < 0.001**
Nonverbal IQ (block design WISC)	109.4 (12.3)	106.9 (12.64)	115.4 (10.8)	0.264
Verbal IQ (similarities WISC)	112.9 (14.9)	111.0 (11.5)	107.5 (13.7)	0.423
**Task performance fMRI**				
Words accuracy (% correct)	92.2 (8.7)	84.5 (17.7)	91.2 (8.8)	**0.004**
Symbols accuracy (% correct)	92.4 (12.6)	89.8 (12.5)	93.9 (9.3)	0.249
Words RT (ms)	1104 (252)	1413 (334)	1233 (294)	** < 0.001**
Symbols RT (ms)	941 (214)	1002 (205)	918 (107)	0.111

Bold values are statistically significant

^a^z-score.

^b^Chi square text, two-sided.

### Ethical approval and guidelines

The study procedures (methods and experimental procedures) were performed in accordance with the relevant guidelines and regulations as approved by the local ethical committees of the three sites (Zurich: “Kantonale Ethikkomission Zürich”; Salzburg: ethical committee of the University of Salzburg; Maastricht: research ethics committee of the Faculty of Psychology and Neuroscience, Maastricht University) and in accordance with the 1964 Helsinki declaration and its later amendments or comparable ethical standards. Children and their legal guardians gave informed consent before participating in the study.

### Procedure

**Figure 1:**
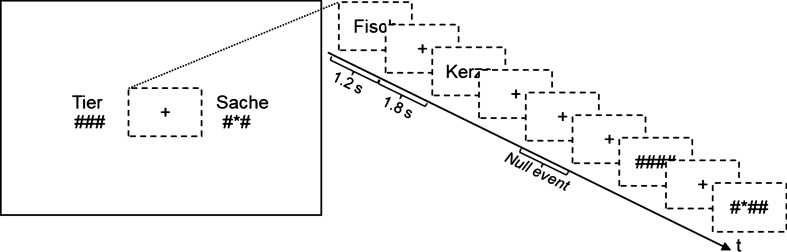
Task. Word or symbol stimuli occurred in the middle of the screen. The captions “Tier”/“###” and “Sache”/“#*#” remained on the screen throughout the experiment, as a reminder of the task: the child was asked to press the left button if a word was an animal (in German “Tier”) or if a string was made up of the same symbols, and to press the right button if a word was not an animal (a thing: in German “Sache”) or if a string contained different symbols. Assignments of left and right buttons were counterbalanced across subjects. Example words are “Fisch” (fish) and “Kerze” (candle).

Either a word or a symbol string was presented every three seconds in the middle of the screen for 1200 ms followed by a fixation cross (1800 ms). The children were asked to press the left button, if the word was an animal or the symbol string was composed of identical symbols, and to press the right button, if the word was a thing, i.e., an inanimate object, or if one of the symbols in the string differed from the other symbols. There were 34 words (all nouns; half animals, half inanimate objects) and 34 symbol strings (half of the strings consisting of hatch marks, half containing a star), which were presented in a pseudo-randomized order. In order to remind the children on the task, the words “animal” and “same” were presented on the left of the screen, and the words “thing” and “different” on the right of the screen constantly throughout the presentation of stimuli and fixation cross (see Fig. 1). The assignment of left and right buttons to the target properties were counterbalanced across subjects. In order to allow a better modelling of the fMRI data 42 null events (fixation cross instead of stimulus) were added.

### fMRI recording and analysis

Across the three sites, data acquisition was kept as similar as possible. The parameters of the T2*-sensitive echo-planar imaging (EPI) sequences, repetition time (TR), number of scans, and voxel size slightly differed between the three sites, to optimize recordings on the particular scanners: In Zurich, fMRI data were acquired on a 3 T GE Healthcare scanner using a T2* sensitive multi-slice echo planar imaging sequence (25 axial slices of 4.6 mm thickness and 0.4 mm gap, TR = 1499 ms, TE = 31 ms, slice resolution = 3.75 mm × 3.75 mm, 64 × 64-pixel matrix, flip angle 50°). In Salzburg, fMRI data were acquired on a 1.5 T Philips Gyroscan NT Scanner (25 axial slices of 5 mm with 0.7 mm gap, TR = 2200 ms, TE = 45 ms, slice resolution = 3.44 mm × 3.44 mm, 64 × 64-pixel matrix, flip angle 90°). In Maastricht, fMRI data were acquired on a 3 T Siemens Allegra scanner (25 axial slices of 5 mm (no gap), TR = 1500 ms, TE = 28 ms, slice resolution = 3.5 mm × 3.5 mm, 64 × 64-pixel matrix, flip angle 90°).

Image processing and statistical analyses were performed jointly for all three sites using SPM12 (v6225) (https://www.fil.ion.ucl.ac.uk/spm) in Zurich. The first few scans were discarded to allow for magnetization equilibration (ZRH/MAS: 4, SBG: 3). Standard preprocessing steps were applied in the following order: Realignment and unwarping, slice time correction, coregistration and segmentation, normalization, resampling (3 × 3 × 3 mm) and smoothing (6 mm FWHM). Normalization to Montreal Neurological Institute (MNI) standard space was done based on the deformations derived by segmentation and a paediatric anatomical template (mean age 9.88 years) created using the Template-OMatic toolbox^70,71^. Movement artefact correction was performed using the ArtRepair toolbox^72^. Based on the scan-to-scan (i.e. framewise) motion threshold of 1.5 mm/TR, volumes exceeding this threshold were repaired using linear interpolation between the nearest unrepaired scans. Out of the 140 analyzed data sets, 32 data sets had at least one volume exceeding the predefined motion threshold (number of repaired volumes for included datasets: mean + SD: 0.8 + 1.9 volumes/data set). Datasets for which more than 5% of the volumes had to be repaired by interpolation (18 datasets excluded out of the initial sample of n = 182) were excluded from all analyses.

A random-effect generalized linear model (GLM) was calculated for each individual, including vectors for both conditions of interest (correctly responded words and symbol strings) as well as incorrectly responded or missed words or symbol stimuli and six movement parameters as vectors of no interest.

The second level analysis focused on print sensitive processing (contrast: words – symbol strings) but the simple contrasts of words or symbols strings vs baseline are illustrated in supplementary Fig. 1 for both groups.

In order to investigate effects of reading fluency on activation patterns, two different strategies were used for our whole brain voxel-wise and the subsequent region of interest (ROI) analyses: First, word reading fluency (z-score) was used as a continuous regressor across all 140 children including dyslexic, typical and intermediate readers. Importantly, this regression approach considers the severity of the reading difficulty. To relate our results to previous group studies we also performed a secondary classical group comparison between the 55 children with dyslexia and the 73 typical readers (as defined in the methods section) using unpaired t-tests. Further we also computed regression analyses with word reading fluency (z-scores) within the group of typical and within the group of dyslexic readers.

**Figure 2:**
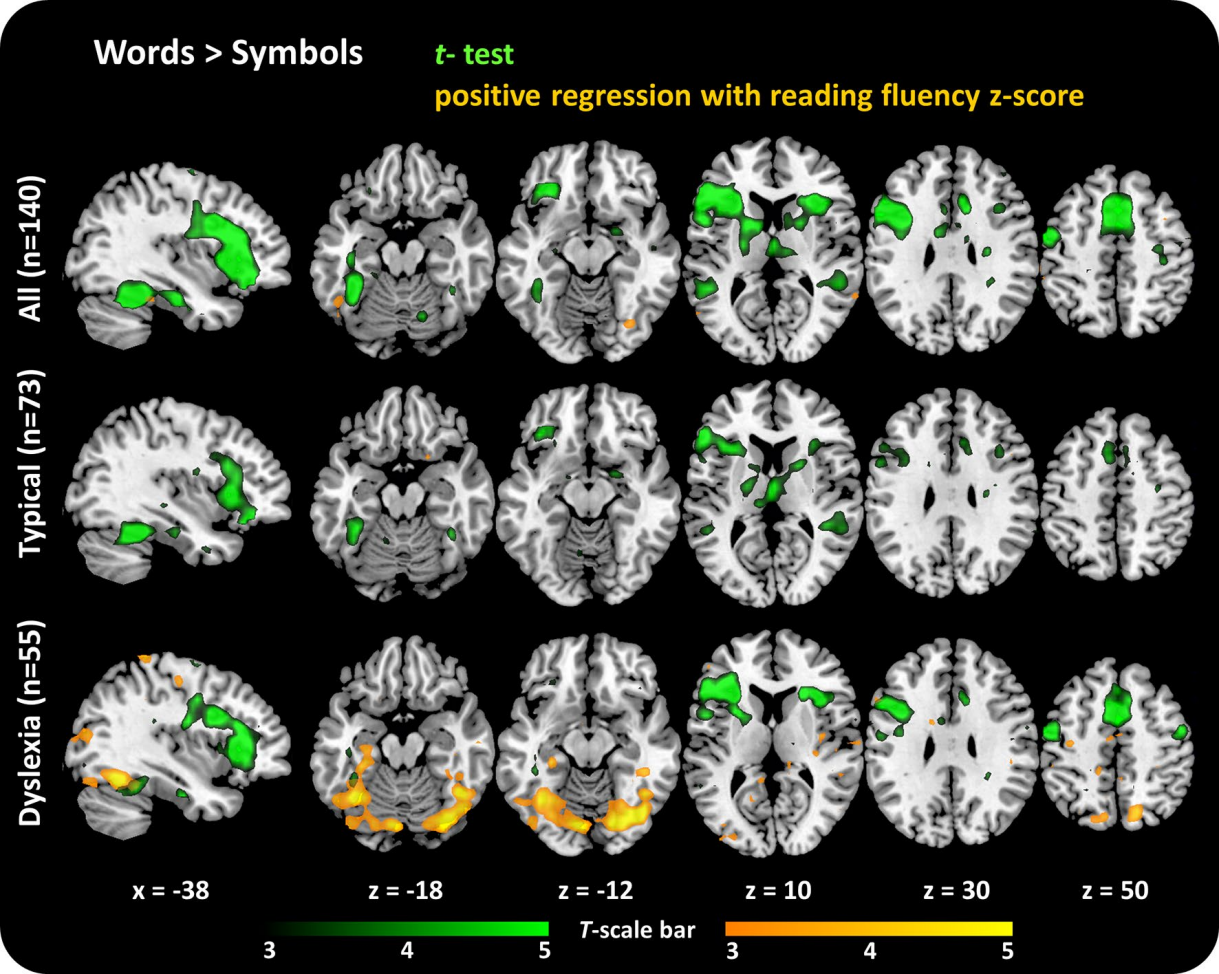
Word-symbol contrasts and regression with reading fluency. Word-sensitive activation in the vOT region was only found for the whole sample and the typical readers but not for children with dyslexia (but see Fig. 3 for direct group contrasts). Typical readers and children with dyslexia both show word-sensitive activation in the left inferior frontal gyrus. The analysis with reading fluency as regressor revealed increased word-sensitive activation the higher childrens’ reading fluency in the left fusiform gyrus in the whole sample on a cluster extent corrected threshold p_(FWEc)_ < 0.05 (p_(CDT)_ < 0.005) and on a voxel wise p_(FWEp)_ < 0.05 in the group of children with dyslexia. No significant correlation was found in the group of typical readers. Illustrated are one sagittal (MNI x = − 38) and five axial slices (MNI z =  −18, z =  − 12, z = 10, z = 30, z = 50). The contrast words > symbols is shown in green and the positive regression of the contrast words > symbols with reading fluency z-scores is shown in orange for each group (all children (top), typical readers (middle) and children with dyslexia (bottom)). Activation threshold for visualization: p_(unc)_ < 0.001 (corresponding to a t > 3.15 for All; t > 3.21 for typical readers and t > 3.25 for dyslexia). The activated clusters are superimposed onto the ch2better.nii template using mricron (https://www.nitrc.org/projects/mricron)^103^.

Voxel-wise results for regression and group analyses are all illustrated on p_(unc)_ < 0.001 (Fig. 2) but only areas surviving voxel wise p_(FWEp)_ correction (and k ≥ 5) or cluster-extent correction (p_(FWEc)_ < 0.05) at a cluster-defining threshold (CDT) of p_(unc)_ < 0.005 are listed in Tables 2, 3 and Table S1 and subsequently discussed. Anatomical labels for maximal voxels in a cluster and subpeaks have been determined using XJView (v8.11) (https://www.alivelearn.net/xjview/) based on the Automated anatomical labelling (AAL) toolbox^73^ and are listed in the corresponding activation Tables 2, 3 and S1.

**Table 2:** Results whole brain analyses.

Typical readers (n = 73)							
Hemisphere	Brain area	(MNI) x, y, z	k	Peak p(FWEc)	Cluster p(FWEp)	T	Z
**Words > symbols**							
L	Inferior frontal gyrus	− 53, 30, 9	17	0	0	6.93	6.03
L	Fusiform gyrus	− 35, − 42, − 21	45	0	0	6.8	5.94
R	Precentral gyrus	46, − 15, 63	10	0.001	0.001	6.29	5.58
L	Inferior frontal gyrus	− 38, 21, 6	21	0	0.003	5.92	5.31
L	Middle frontal gyrus	− 35, 33, − 12	8	0.002	0.003	5.9	5.3
	Undef	1, − 12, 9	6	0.004	0.004	5.78	5.21
L	Medial frontal gyrus	− 11, − 3, 60	5	0.005	0.006	5.72	5.16
R	Caudate	22, 9, 15	5	0.005	0.018	5.38	4.9
L	Superior temporal gyrus	− 53, − 42, 6	5	0.005	0.022	5.33	4.86
**Symbols > words**							
	None						
**Dyslexia (n = 55)**							
Words > symbols							
L/R	Superior/medial frontal gyrus	− 2, 3, 60	147	0	0	9.86	7.37
L	Inferior frontal gyrus	− 35, 30, 9	245	0	0	9.34	7.12
L	Undef	− 11, − 6, 24	5	0.003	0	7.44	6.11
R	Inferior frontal gyrus	34, 24, 6	44	0	0	7.19	5.97
L	Precentral gyrus	− 53, − 9, 45	31	0	0.001	6.6	5.6
L	Cerebellum	− 11, − 45, − 33	15	0	0.001	6.53	5.55
R	Cerebellum	34, − 54, − 30	12	0	0.001	6.39	5.47
R	Precentral gyrus	49, − 6, 48	12	0	0.002	6.26	5.38
L	Putamen/insula	− 26, 9, 12	5	0.003	0.004	6.1	5.28
R	Caudate	25, 24, 9	6	0.002	0.01	5.84	5.1
**Symbols > words**							
R	Parahippocampal gyrus	28, − 48, − 6	7	0.001	0.008	5.89	5.13

For all contrasts the significance level at whole-brain peak-level threshold p_(FWEp)_ < 0.05, k ≥ 5. Labels of brain regions determined using the XJView (AAL atlas); k: cluster size; R: right; L: left.

**Table 3:** Results whole brain analyses, regressions and group contrasts.

Regressions with reading fluency z- scores								
Hemisphere	Brain area		(MNI) x, y, z	k	Peak p(FWEc)	Cluster p(FWEp)	T	Z
**All (n = 140)**								
Words > symbols, positive regression with reading fluency z-score^#^								
L	Fusiform gyrus^**#**^		− 44, − 36, − 21	186	0.021	0.692	3.89	3.78
Words > symbols, negative regression with reading fluency z-score^#^								
	None^#^							
**Typical readers (n = 73)**								
Words > symbols, positive regression with reading fluency z-score^#^								
	None^#^							
Words > symbols, negative regression with reading fluency z-score^#^								
	None^#^							
**Dyslexia (n = 55)**								
Words > symbols, positive regression with reading fluency z-score								
L	Fusiform gyrus		− 38, − 57, − 15	8	0.001	0.001	6.42	5.47
R	Fusiform gyrus/middle occipital gyrus		40, − 69, − 12	29	0	0.001	6.4	5.46
Words > symbols, negative regression with reading fluency z-score^#^								
	None^#^							
**Group contrasts**								
Dyslexia > typical readers								
Words > symbols^#^								
L	Precentral gyrus^#^		− 53, − 21, 42	264	0.001	0.239	4.42	4.25
R	Precentral gyrus^#^		61, − 6, 24	172	0.015	0.711	3.95	3.82

For all contrasts the significance level at whole-brain peak-level threshold p_(FWEp)_ < 0.05, k ≥ 5 except for contrasts designated with^#^ are reported on cluster-level threshold p_(FWEc)_ < 0.05 at cluster-defining threshold at p_(CDT)_ < 0.005. Labels of brain regions were determined using the XJView (AAL atlas); k: cluster size; R: right; L: left.

In addition, literature-based ROI analyses (spheres, radius = 4 mm) were computed for the left visual WFA (MNI: − 46, − 52, − 2) and the left LFA (MNI: − 40, − 78, − 18)^23^. The mean beta values of these ROIs were extracted with SPM12 and entered into linear mixed model analyses (LMM) using SAS® 9.4 (procedure PROC MIXED) including the fixed factors *ROI* (WFA, LFW), *condition* (words, symbols) and *reading fluency* (continuous measure or groups: dyslexia, typical readers) and random factors *site* (SBG, ZRH, MAS) and *subject* as well as the covariate *age*. To account for possible differences between sites, we corrected for site effects either by using effects coding (whole brain analyses)^74,75^ or by including *site* as a random factor in our LMM. For LMM analyses, studentized conditional residuals were computed to identify and exclude potential outliers and in order to correct for variance inhomogeneity. An outlier cutoff of three standard deviations from the mean was used for all analyses^76^. In addition, QQ-plots were inspected to ensure the assumption of normality and homoscedasticity of predicted versus conditional residual plots. All reported p-values of post hoc analyses are Tukey–Kramer corrected.

### Behavioral analysis

Accuracy (Acc) and reaction time (RT) of the in-scanner task were analysed using SAS^®^ 9.4 (Cary, NC: SAS Institute Inc.; https://www.sas.com/en_us/software/sas9.html) using two types of models: (1) with a within subject factor *condition* (words vs. symbols) and *reading fluency* as a covariate of interest for the whole sample (including the intermediate readers) and, (2) with a within subject factor *condition* (words vs. symbols) and a between subject factor *group* (dyslexic vs. typical readers; intermediate readers excluded). For accuracy, a beta regression model (proc glimmix) has been used to account for the distribution of the values in the standard unit interval (0, 1). For reaction time the function Proc Mixed was applied. Both models included the random intercepts for *subject* and *site*.

## Results

### Behavioral results

On average, accuracy in the fMRI task was very high (mean > 84% correct in all groups and both conditions, see Table 1). The more fluently a child was reading, the higher the accuracy was in the fMRI task (*reading fluency*, F(1,124) = 9.73, p < 0.0022), irrespective of *condition* (*reading fluency x condition*, F(1,124) = 0.27, p = 0.6075). Symbol strings were responded to more accurately (Accuracy: *condition*, F(1,124) = 18.31, p < 0.0001) and faster (RT: *condition*, F(1,129) = 230.33; p < 0.0001) than words. This condition-sensitive slowing was more pronounced, the less fluent a child was reading (*reading fluency x condition*, F(1,129) = 44.35, p < 0.0001). This interaction modulated the main effect of *reading fluency* on RT (*reading fluency*, F(1,129) = 17.24, p < 0.0001).

These behavioural effects were also reflected in the groupwise analysis: At the generally high performance level, children with typical reading skills were more accurate than children with dyslexia (*group*, F(1,112) = 4.75, p = 0.0314), but irrespective of *condition* (*group x condition*, F(1,112) = 0.27, p = 0.6033). Accuracy was higher for symbol strings than words (*condition*, F(1,112) = 22.14, p < 0.0001) and the children, responded more slowly to words than to symbol strings (*condition*, F(1,118) = 379.99; p < 0.001). This reaction time difference was more pronounced in the children with dyslexia than in the typical reading children resulting in an interaction (*group* × *condition*, F(1,118) = 46.54, p < 0.0001) that also modulated the main effect of *group* (F(1,118) = 14.34, p < 0.0002). Post-hoc t-tests showed that children with dyslexia had slower reaction times (p < 0.001) and lower accuracies (p = 0.004) for words than typical reading children but the groups did not differ in their performance on symbol strings.

### fMRI whole brain results

#### Print-sensitive activation

Both children with typical reading skills and children with dyslexia showed word-sensitive activation (words > symbol strings) in left inferior and medial frontal regions and parts of the basal ganglia (p_(FWEp)_ < 0.05, Fig. 2, Table 2). Word-sensitive activation in the left fusiform and superior temporal gyri was only found for the typical readers and not the children with dyslexia.

The contrasts words vs baseline or symbols vs baseline showed activation in both groups in bilateral ventral occipito-temporal regions and in left precentral regions. Words additionally showed bilateral middle and inferior frontal activation. For a detailed description of activated cluster per group and condition see supplementary Fig. 1.

#### Regression of print-sensitive processing with reading fluency

Only the left fusiform gyrus showed a significant relation of word-sensitive activation with reading ability, in the form of increased functional activation with increased reading performance (Table 3, Fig. 2; p_(FWEc)_ < 0.05, cluster size-corrected at CDT p_(unc)_ < 0.005). Interestingly, separate regression analyses by group for the a) typical readers and b) the children with dyslexia showed an increase in the functional activation of bilateral fusiform gyri (FFG) with reading fluency (p_(FWEc)_ < 0.05) in the children with dyslexia only.

No significant (p_(FWEc)_ < 0.05) negative correlation with reading fluency and print-sensitive activation was found neither for the whole sample, nor for any of the two reading groups (Table 3).

#### Group effects on print sensitive activation

**Figure 3:**
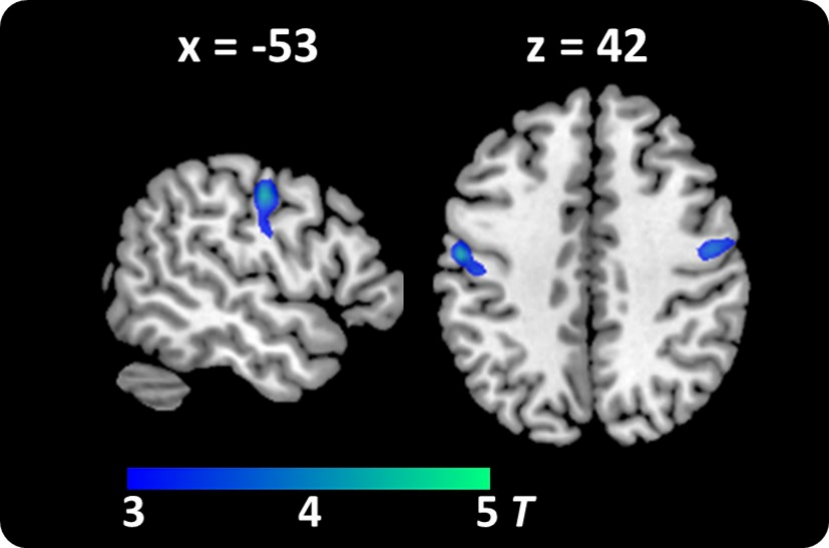
Group contrast children with dyslexia vs. children with typical reading skills. Higher word-sensitive (words—symbols) activation for children with dyslexia than for typical readers was found in bilateral precentral gyri. Activation threshold for visualization is set to p_(unc)_ < 0.001 (corresponding to t > 3.16). The activation clusters are superimposed onto the ch2better.nii template using mricron (https://www.nitrc.org/projects/mricron)^103^.

The group of typical readers did not show any region with increased word-sensitive activation compared to the children with dyslexia surviving a cluster extent (or voxel-wise) correction. On the contrary, bilateral precentral gyri showed enhanced activation for print-sensitive activation in children with dyslexia compared to typical readers (Table 3, Fig. 3; p_(FWEc)_ < 0.05, cluster size-corrected at CDT p_(unc)_ < 0.005).

### Left vOT region of interest (ROI) analyses

#### Impact of reading fluency on print-sensitive processing in the left vOT

**Figure 4:**
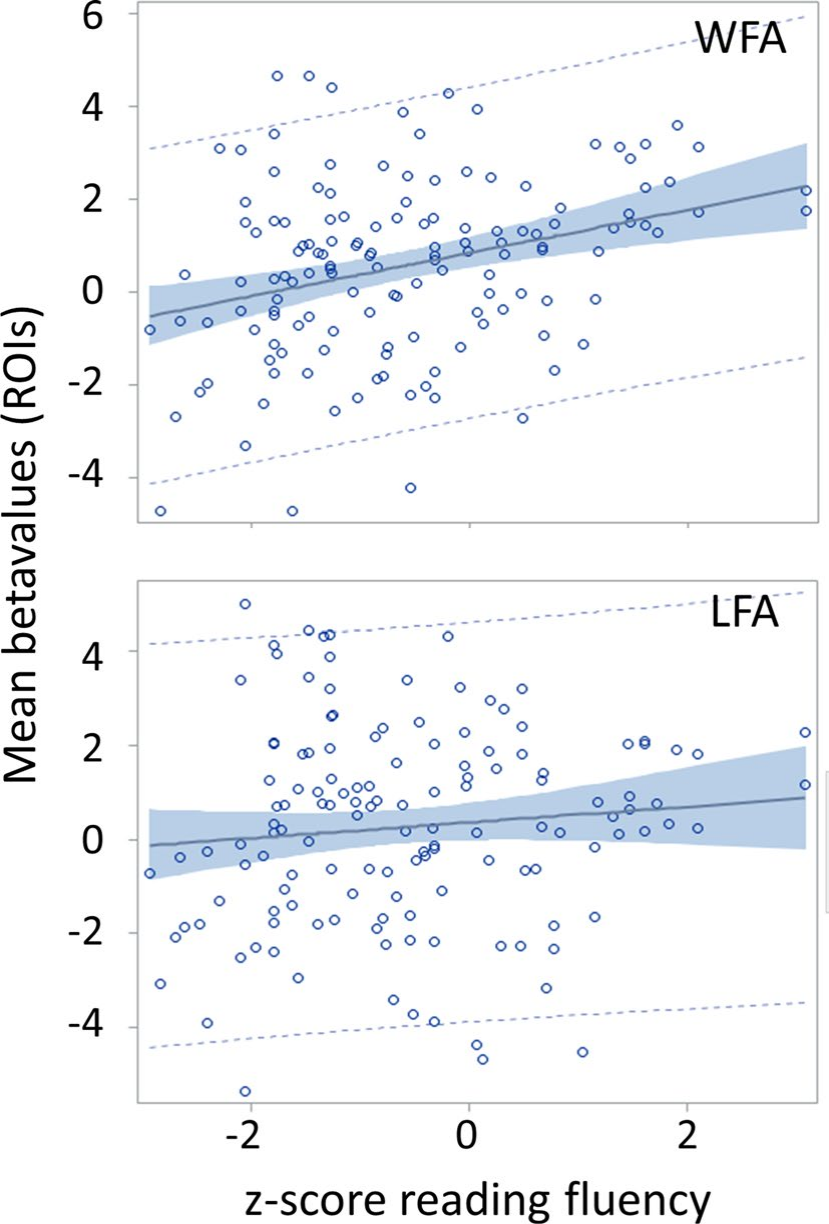
Regression plots showing the positive association between reading fluency and print sensitive activation (words-symbols) in the WFA ROI (top: F(1,137) = 15.09, p = 0.0002, r^2^ = 0.0992)) and the LFA ROI (bottom: F(1,135) = 1.37, p = 0.2433, r^2^ = 0.0101) of the entire sample (including gap group; n = 140). The blue shaded areas represent the 95% confidence limits, the blue dashed lines the 95% prediction limits.

When using word reading fluency score as a covariate of interest for the whole sample of 140 children, a trend for the triple interaction of *reading fluency, ROI (WFA, LFA)* and *condition (Words, Symbols)* (F(1,411) = 3.53, p = 0.0610), a significant interaction of *reading fluency* and *condition* (F(1,411) = 6.66, p = 0.0102) and a trend for *ROI x condition* (F(1,411) = 3.1, p = 0.0790) was found. In addition, main effects of *ROI* (F(1,411) = 40.42, p = 0.0001) and *condition* (F(1,411) = 12.22, p = 0.0005) were also significant. The significant regression of print-sensitive activation with *reading fluency* in the left WFA and the nonsignificant regression with left LFA ROIs are illustrated in Fig. 4, for supplementary site-wise analyses, please refer to the supplementary analysis section.

Because of the significant association of reading fluency and print sensitive processing in left VOT in children with dyslexia but not in typical reading children as revealed in the whole brain analyses we repeated the same model as described above separately for the group of typical readers and for children with dyslexia. Both groups showed a significant (typical) or trend (dyslexia) main effect of *ROI* (typical readers: (F(1,211) = 21.85, p = 0.0001; dyslexia (F(1,157) = 3.78, p = 0.0535)) and an interaction of *condition* and *reading fluency* (typical readers: (F(1,211) = 6.70, p = 0.0103; dyslexia (F(1,157) = 18.11, p < 0.0001)). The *condition* main effect was only significant in children with dyslexia (typical readers: (F(1,211) = 0.99, p = 0.3197; dyslexia (F(1,157) = 19.97, p < 0.0001)), while only typical readers showed an interaction of *ROI* and *condition* (typical readers: (F(1,211) = 4.23, p = 0.0408; dyslexia (F(1,157) = 2.10, p = 0.1495)), indicating a more focal print sensitivity effect in the classic WFA in typical readers as compared to children with dyslexia.

#### Group differences in print-sensitive vOT activation

**Figure 5:**
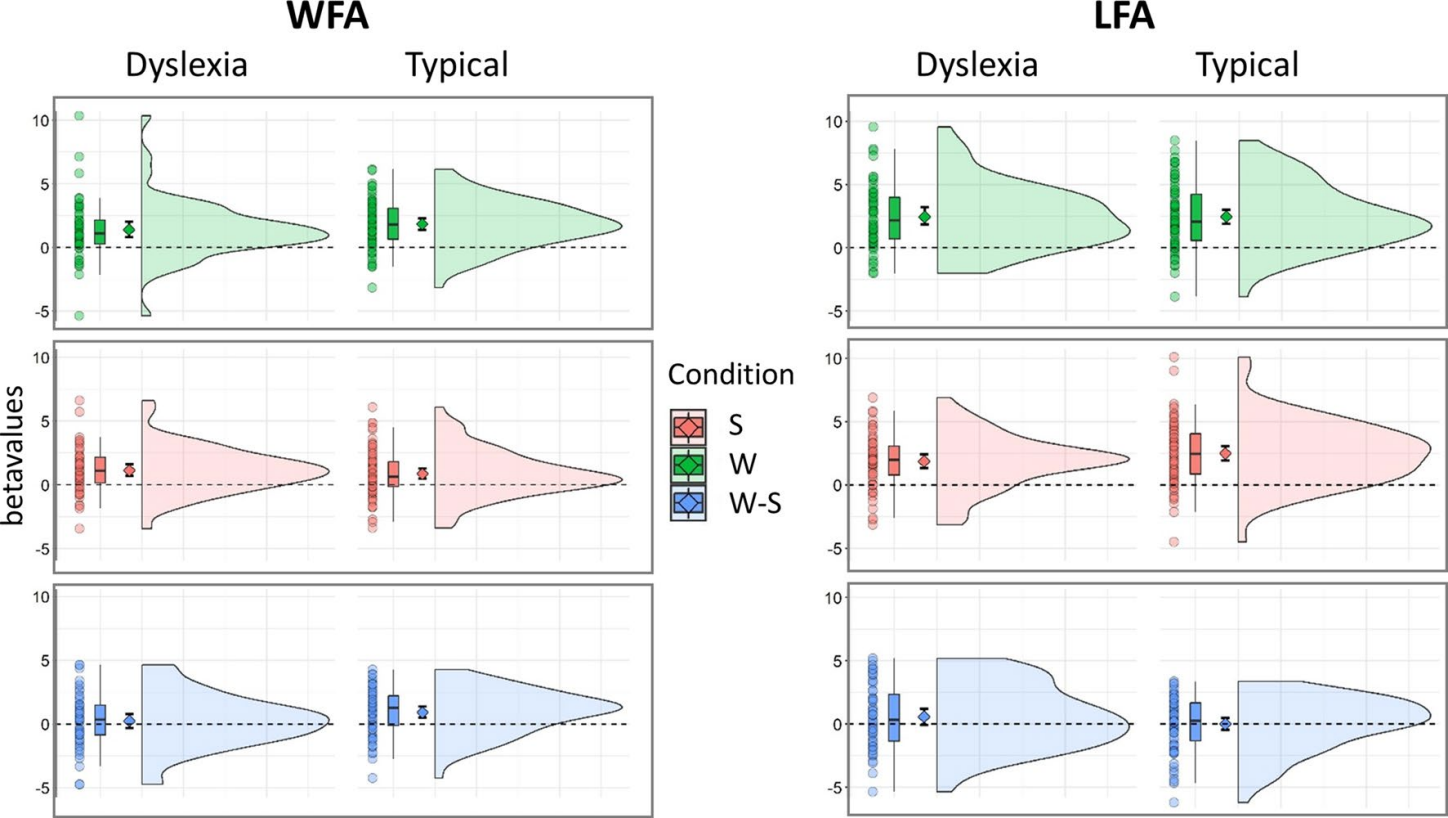
Mean betavalues for ROI data. The plots illustrate the betavalues for children with dyslexia and typical reading children for each condition; Words (green), Symbols (red), and the Words-Symbols difference (blue). The ROIs WFA and LFA are shown separately in the left and right columns, respectively. Each 'raincloud' plot shows (from left to right) individual data points, boxplot with median and interquartile range, mean and 95% CI, and sample distribution.

Activation in the vOT ROIs corrected for age and site was stronger for words than for symbol strings (*condition*, F(1,374) = 42.6, p < 0.001) and overall activation in the LFA was stronger than activation in the WFA (*ROI*, F(1,374) = 8.2, p = 0.0044). The word-sensitive activation was stronger in typical readers than in children with dyslexia in the WFA but not in the LFA (*group x condition x ROI*, F(1,374) = 4.66, p = 0.0316, c.f. Fig. 5). The *group* main effect was not significant (*group*, F(1,374) = 0.52, p = 0.472). Posthoc t-tests showed that only the typical readers had a more pronounced BOLD signal to words than symbol strings (print sensitive processing) in the WFA (t = 3.37, p = 0.0189) and more activation to symbol strings in the LFA than the WFA (t = − 5.63, p =  < 0.001). Children with dyslexia showed more pronounced activation in the LFA than WFA to words (t = − 3.20, p = 0.0323) c.f. Fig. 5. Two additional categorical analyses included also the small group of intermediate reading children (n = 12) or an extended intermediate reader group (n = 29) in the models. These LMMs largely confirmed the results of the main model with the two core groups and are added in the supplementary material.

## Discussion

Neuroimaging studies and a growing number of meta-analyses in the past years consistently reported alterations in the microstructure and in activation of the left ventral occipito-temporal cortex during reading-related tasks in poorly reading children and adults as compared with typical readers^41–43,64,77,78^. With this multicentre study we aimed to confirm the findings of a hypoactive vOT system in poor readers in an exceptionally large sample (n = 140) of children spanning the whole spectrum of reading skills from very poor (dyslexic) to highly fluent readers. Specifically, we examined functional activation differences with a reading for meaning and a symbol string comparison task to clarify how print-sensitive processing in the left vOT is related to reading fluency. We took advantage of our large sample to conduct both continuous and categorical (group comparison) analyses. The results of the whole brain and the ROI analyses revealed two key findings: First, a nonlinear association of print-sensitive activation in left vOT with reading scores, which is especially pronounced in the poor reading range but less in the typical reading range. And second, a more focal print-sensitive activation in the WFA in children performing within the typical reading range as compared with poorly reading children.

Continuous whole-brain analyses in the full sample revealed a positive association of print-sensitive activation with reading fluency in only one distinct cluster over the whole brain located in the centre of the visual word form system of the left midfusiform gyrus: the poorer a child was reading, the lower was his/her print-sensitive activation in this region. This is in agreement with many previous studies showing the critical role of this region for reading and dyslexia (see for example meta-analyses^41–43^).

The current findings, however, extend those from previous studies by showing that the relation between left vOT activation and reading skills is neither linear across the entire range of reading nor dependent on classical thresholds for impaired and unimpaired reading. Despite the large sample of typical readers and their large variation in reading scores, no relation of reading fluency to activation in the left vOT survived activation thresholds in the voxel-wise analysis in this group. Children with dyslexia, however, showed a strong positive association between their reading fluency and print-sensitive processing in extended and bilateral regions including the fusiform gyri. This suggests that word-sensitive activation decreases mainly among the children with dyslexia with increasing severity of their reading difficulties, but not among the typical readers. Importantly the absence of this brain-behaviour association cannot be explained by reduced variation in the reading fluency of typical readers.

Our group wise analyses provided more details to explain this finding. First, no print-sensitive activation in the fusiform gyri was detected in the group of children with dyslexia. Together with the findings of a very strong positive association with reading skills, this result thus suggests a very high variability in the print-sensitive response of the vOT in the group of poor readers that is partly explained by severity. Second, children with typical reading skills showed strong print-sensitive activation in the left fusiform gyrus. Together with the absent modulation of print-sensitive activation through reading skills, our data indicate a minor impact of reading fluency on print-sensitive activation in the typical reading range. Moreover, the direct group contrast of print-sensitive processing, showed only a stronger involvement of bilateral precentral gyri in children with dyslexia but did not yield any differences in the activation of the vOT. These results indicate that a certain basic level of print-sensitive processing in the left vOT is a prerequisite for reading skills in the average performance range. And while the individual variability of reading fluency in the typical range in school children seems to be only weakly related to the level of print-sensitive vOT activity, the variability in reading performance in the very poor performance range shows a strong and linear association with the vOT sensitivity to print.

Our findings extend the results of two recent studies comparing the print-sensitivity effect in younger children^30^ or print, symbol, falsefont and digit string processing in somewhat older (~ 11.5 years) children^79^. While no group difference was detected for the print-sensitivity effects in these studies, clear and unspecific hypoactivation in the left vOT was reported for processing words, symbol and digit strings^30,79^ suggesting a more general visual object processing failure in children with dyslexia. In contrast to these studies the activation to symbol strings did not differ between groups in our study as shown in the ROI analyses of the WFA and LFA. Instead, a distinct reduction of the print sensitivity effect in the WFA in children with dyslexia thus suggested a more focal modulation of print-sensitive processing in the WFA of typical readers. While a failure to detect group effects in the left vOT in many previous studies could have been due to the rather small group sizes, or the often rather lenient criteria to categorize children with dyslexia this is unlikely the case for the current study which included more than 50 children per group and only severely impaired children formed the dyslexia group. More likely, based on our results, is the assumption that children with more severe forms of reading disability show a larger failure in print-sensitive processing in the left vOT which shows up also in simple tasks, while children close to average reading scores hardly differ anymore in the functional activation of this system during simple implicit reading and semantic judgment tasks. This is also corroborated by the performance data in our fMRI tasks. Even though typical readers performed more accurately and faster, the overall performance in this task was very good also in the group of children with dyslexia. While it was our aim, to implement a task that can be done by all children, more challenging tasks, may have resulted in more pronounced group differences also regarding the activation of the VWFA.

Previous studies showed that, deficient VWFA function may be explained by a basic deficit in learning grapheme-phoneme correspondences in children with dyslexia^80,81^. Deficient activation may become more accentuated also in poor and intermediate readers when specifically challenging the system with tasks that emphasize phonological processing^22,45,52–56,62,63,82^. How much the left vOT is engaged in visual word processing may thus not only depend on the reading skill level of a child, but could also be influenced by differential deployment of top-down recruitment with task requirements^9^. Like in other studies, applying region of interest analyses to specifically examine the activation pattern of the visual word form area yielded focal print-sensitive activation differences between groups. Connectivity studies show, that the VWFA development^13,29^, and its function are largely constrained and predicted by its privileged connectivity to various areas within the (temporal) language network but also its tight coupling to the dorsal fronto-parietal attention network^51,83,84^. Such connectivity findings suggest that the VWFA activation is tuned by input from higher order language areas and attentional processes, depending also on the task requirements, the reading strategy and experience of the children. The extended connectivity of the left vOT thus seems a prerequisite for guiding the emerging specialization in the VWFA^13^ which is initiated within the first months of formal reading acquisition through the learning of letter and speech sound associations in alphabetic languages^26,27,29,33,85,86^. In addition the strong structural and functional link of the VWFA to the attentional system^51,87,88^ may support the guidance of visual attention by amplifying the representations of words and fostering subsequent phonological and lexical processing^51^. Impairments in attentional mechanisms in dyslexia may cause poor orthographic representations^89^ and phonological decoding deficits^90^. Importantly targeting these core systems through the application of phonics training enhancing grapheme-phoneme correspondences^91,92^ or also attentional training with action video games^93,94^ and reading acceleration^95^ may support poor readers.

The strong group difference of increased (rather than decreased) print-sensitive activation in children with dyslexia in left and right precentral regions is well in line with previous findings. Such overactivation has been reported consistently across studies [e.g.^30,96^] and meta-analyses^41,42^. A recent meta-analysis specifically addressing word processing in shallow and deep orthographies indicated that this overactivation is especially prominent in shallow orthographies^44^. Accordingly, the hyperactivation observed in our sample of children with reading difficulties may have been especially pronounced because of using simple and short words in our task, which can be read easily and automatically by typical but not by children with reading difficulties. The precentral regions are known to be involved in the articulatory network^97^, speech production^98^ and silent articulation^99^. An overlap of frontal regions usually showing hyperactivation in individuals with reading disorder with regions supporting articulation has been demonstrated in a recent quantitative meta-analysis and thereby supports the notion that such hyperactivation reflects compensatory processing to access the meaning of print^96^. Thus, poor reading children may use these regions to support reading by employing letter-by-letter decoding or covert articulation processes^30^. As an alternative explanation the hyperactivation in the precentral gyri could also reflect greater difficulties with and increased resources necessary for covert articulation or general processing of words in children with dyslexia. In either case these explanations converge with less efficient processing as indicated by slower reaction times to semantic decisions on words (and not symbols strings) in our task.

In order to achieve a large sample size, we collected data across three different sites, including two different languages with similar semi-transparent^100,101^ orthographic complexity (Dutch and German). This merging of three different groups may be seen as a limitation of this study. To address this potential limitation, our linear mixed models included site and age as covariates to regress out potential confounds. This suggests that the critical effects in this study were not affected by the different conditions across the three sites or by the differences in age among the children, and thus generalize across involved languages and educational systems. Further one needs to keep in mind, that the present study design does not allow to finally conclude on whether the hypoactivation of the VWFA and the hyperactivation in the precentral gyri to print reflect specific dyslexia effects or whether these differences may rather be explained by differences in print exposure or reading level between children with dyslexia and typical reading skills. However, a study comparing adolescents with dyslexia to age- and reading-level matched control groups, suggested that the hypoactivation in the left parietal and fusiform gyri indeed reflect atypical brain function in dyslexic individuals, while the frontal hyperactivation is more likely related to the current reading skills independent of dyslexia^102^.

Taken together, this large-scale neuroimaging study on the influence of word reading fluency on brain activation emphasizes the important role of the left vOT for reading skills and highlights its functional impairment in poor reading children. It extends the insight of previous large-scale studies specifically challenging phonological processing and using more liberal criteria for defining dyslexia by focussing on reading for meaning. Thus, the present study represents one of the largest datasets (n = 140) on functional activation in children with severe dyslexia compared to typical readers and clearly corroborates previous notions that the print-sensitive activation in the left vOT represents a critical limiting factor of reading for meaning especially at the lower end of the reading fluency spectrum. Given that the most robust underactivation in children with dyslexia occurred in this region, the left vOT function appears to be a prime target for supportive interventions in those needing it most.

## Supplementary information

Supplementary Information.

## Data Availability

The data that support the findings of this study are available but restrictions apply to the availability of these data, due to the restricted and site-specific consent of research participants. Data are however available from the authors upon reasonable request and with permission of the principal investigator of each site.

## References

[1] Peters L, Ansari D (2019). Are specific learning disorders truly specific, and are they disorders?. Trends Neurosci. Educ..

[2] Shaywitz SE, Shaywitz BA, Fletcher JM, Escobar MD (1990). Prevalence of reading disability in boys and girls. Results of the Connecticut Longitudinal Study. JAMA.

[3] Bradley L, Bryant PE (1978). Difficulties in auditory organisation as a possible cause of reading backwardness. Nature.

[4] Baker CI (2007). Visual word processing and experiential origins of functional selectivity in human extrastriate cortex. Proc. Natl. Acad. Sci. USA.

[5] Centanni TM (2018). Early development of letter specialization in left fusiform is associated with better word reading and smaller fusiform face area. Dev. Sci..

[6] Coch D, Meade G (2016). N1 and P2 to words and wordlike stimuli in late elementary school children and adults. Psychophysiology.

[7] Cohen L (2000). The visual word form area: Spatial and temporal characterization of an initial stage of reading in normal subjects and posterior split-brain patients. Brain.

[8] Cohen L (2002). Language-specific tuning of visual cortex? Functional properties of the Visual Word Form Area. Brain.

[9] Dehaene S, Cohen L (2011). The unique role of the visual word form area in reading. Trends Cogn. Sci..

[10] Glezer LS, Jiang X, Riesenhuber M (2009). Evidence for highly selective neuronal tuning to whole words in the "visual word form area". Neuron.

[11] McCandliss BD, Cohen L, Dehaene S (2003). The visual word form area: Expertise for reading in the fusiform gyrus. Trends Cogn. Sci..

[12] Price CJ, Moore CJ, Frackowiak RS (1996). The effect of varying stimulus rate and duration on brain activity during reading. Neuroimage.

[13] Saygin ZM (2016). Connectivity precedes function in the development of the visual word form area. Nat. Neurosci..

[14] Vinckier F (2007). Hierarchical coding of letter strings in the ventral stream: Dissecting the inner organization of the visual word-form system. Neuron.

[15] Turkeltaub PE, Gareau L, Flowers DL, Zeffiro TA, Eden GF (2003). Development of neural mechanisms for reading. Nat. Neurosci..

[16] Price CJ, Devlin JT (2003). The myth of the visual word form area. Neuroimage.

[17] Vogel AC, Petersen SE, Schlaggar BL (2014). The VWFA: It's not just for words anymore. Front. Hum. Neurosci..

[18] Brem S (2006). Evidence for developmental changes in the visual word processing network beyond adolescence. Neuroimage.

[19] Mano QR (2013). The role of left occipitotemporal cortex in reading: Reconciling stimulus, task, and lexicality effects. Cereb. Cortex.

[20] Kronschnabel J, Schmid R, Maurer U, Brandeis D (2013). Visual print tuning deficits in dyslexic adolescents under minimized phonological demands. Neuroimage.

[21] Vogel AC, Petersen SE, Schlaggar BL (2012). The left occipitotemporal cortex does not show preferential activity for words. Cereb. Cortex.

[22] van der Mark S (2009). Children with dyslexia lack multiple specializations along the visual word-form (VWF) system. Neuroimage.

[23] Thesen T (2012). Sequential then interactive processing of letters and words in the left fusiform gyrus. Nat. Commun..

[24] Lerma-Usabiaga G, Carreiras M, Paz-Alonso PM (2018). Converging evidence for functional and structural segregation within the left ventral occipitotemporal cortex in reading. Proc. Natl. Acad. Sci. USA.

[25] Bouhali F, Bézagu Z, Dehaene S, Cohen L (2019). A mesial-to-lateral dissociation for orthographic processing in the visual cortex. Proc. Natl. Acad. Sci..

[26] Brem S (2010). Brain sensitivity to print emerges when children learn letter-speech sound correspondences. Proc. Natl. Acad. Sci. USA.

[27] Maurer U (2006). Coarse neural tuning for print peaks when children learn to read. Neuroimage.

[28] Ben-Shachar M, Dougherty RF, Deutsch GK, Wandell BA (2011). The development of cortical sensitivity to visual word forms. J. Cogn. Neurosci..

[29] Dehaene-Lambertz G, Monzalvo K, Dehaene S (2018). The emergence of the visual word form: Longitudinal evolution of category-specific ventral visual areas during reading acquisition. PLoS Biol.

[30] Chyl K (2018). Prereader to beginning reader: Changes induced by reading acquisition in print and speech brain networks. J. Child Psychol. Psychiatry.

[31] Dehaene S, Cohen L, Sigman M, Vinckier F (2005). The neural code for written words: A proposal. Trends Cogn. Sci..

[32] Zhao J (2014). Fine neural tuning for orthographic properties of words emerges early in children reading alphabetic script. J. Cogn. Neurosci..

[33] Pleisch G (2019). Simultaneous EEG and fMRI reveals stronger sensitivity to orthographic strings in the left occipito-temporal cortex of typical versus poor beginning readers. Dev. Cogn. Neurosci..

[34] Pleisch G (2019). Emerging neural specialization of the ventral occipitotemporal cortex to characters through phonological association learning in preschool children. Neuroimage.

[35] Xue G, Chen C, Jin Z, Dong Q (2006). Language experience shapes fusiform activation when processing a logographic artificial language: An fMRI training study. Neuroimage.

[36] Martin L (2019). The VWFA is the home of orthographic learning when houses are used as letters. eneuro.

[37] Li J, Osher DE, Hansen HA, Saygin ZM (2019). Cortical selectivity driven by connectivity: Innate connectivity patterns of the visual word form area. bioRxiv.

[38] Glezer LS, Riesenhuber M (2013). Individual variability in location impacts orthographic selectivity in the "visual word form area". J. Neurosci..

[39] Bruno JL, Zumberge A, Manis FR, Lu ZL, Goldman JG (2008). Sensitivity to orthographic familiarity in the occipito-temporal region. Neuroimage.

[40] Sandak R, Mencl WE, Frost SJ, Pugh KR (2004). The neurobiological basis of skilled and impaired reading: Recent findings and new directions. Sci. Stud. Read..

[41] Richlan F, Kronbichler M, Wimmer H (2009). Functional abnormalities in the dyslexic brain: A quantitative meta-analysis of neuroimaging studies. Hum. Brain Mapp..

[42] Richlan F, Kronbichler M, Wimmer H (2011). Meta-analyzing brain dysfunctions in dyslexic children and adults. Neuroimage.

[43] Maisog JM, Einbinder ER, Flowers DL, Turkeltaub PE, Eden GF (2008). A meta-analysis of functional neuroimaging studies of dyslexia. Ann. N. Y. Acad. Sci..

[44] Martin A, Kronbichler M, Richlan F (2016). Dyslexic brain activation abnormalities in deep and shallow orthographies: A meta-analysis of 28 functional neuroimaging studies. Hum. Brain. Mapp..

[45] Paulesu E (2001). Dyslexia: Cultural diversity and biological unity. Science.

[46] Siok WT, Perfetti CA, Jin Z, Tan LH (2004). Biological abnormality of impaired reading is constrained by culture. Nature.

[47] Pflugshaupt T (2009). About the role of visual field defects in pure alexia. Brain.

[48] Hirshorn EA (2016). Decoding and disrupting left midfusiform gyrus activity during word reading. Proc. Natl. Acad. Sci. USA.

[49] Dehaene S, Cohen L, Morais J, Kolinsky R (2015). Illiterate to literate: Behavioural and cerebral changes induced by reading acquisition. Nat. Rev. Neurosci..

[50] Price CJ, Devlin JT (2011). The interactive account of ventral occipitotemporal contributions to reading. Trends Cogn. Sci...

[51] Chen L (2019). The visual word form area (VWFA) is part of both language and attention circuitry. Nat. Commun..

[52] Brambati SM (2006). Neuropsychological deficits and neural dysfunction in familial dyslexia. Brain Res..

[53] Richlan F (2010). A common left occipito-temporal dysfunction in developmental dyslexia and acquired letter-by-letter reading?. PLoS ONE.

[54] Wimmer H (2010). A dual-route perspective on poor reading in a regular orthography: An fMRI study. Cortex.

[55] Shaywitz BA (2002). Disruption of posterior brain systems for reading in children with developmental dyslexia. Biol. Psychiatry.

[56] Cao F, Bitan T, Chou TL, Burman DD, Booth JR (2006). Deficient orthographic and phonological representations in children with dyslexia revealed by brain activation patterns. J. Child Psychol. Psychiatry.

[57] Meyler A (2007). Brain activation during sentence comprehension among good and poor readers. Cereb. Cortex.

[58] Kronbichler M (2006). Evidence for a dysfunction of left posterior reading areas in German dyslexic readers. Neuropsychologia.

[59] Schulz E (2008). Impaired semantic processing during sentence reading in children with dyslexia: Combined fMRI and ERP evidence. Neuroimage.

[60] Maurer U (2010). The development of print tuning in children with dyslexia: Evidence from longitudinal ERP data supported by fMRI. Neuroimage.

[61] McCrory EJ, Mechelli A, Frith U, Price CJ (2005). More than words: A common neural basis for reading and naming deficits in developmental dyslexia?. Brain.

[62] Tanaka H (2011). The brain basis of the phonological deficit in dyslexia is independent of IQ. Psychol. Sci..

[63] Shaywitz BA (2007). Age-related changes in reading systems of dyslexic children. Ann. Neurol..

[64] Jednorog K (2015). How reliable are gray matter disruptions in specific reading disability across multiple countries and languages? insights from a large-scale voxel-based morphometry study. Hum. Brain Mapp..

[65] Bach S, Richardson U, Brandeis D, Martin E, Brem S (2013). Print-specific multimodal brain activation in kindergarten improves prediction of reading skills in second grade. Neuroimage.

[66] Tewes U, Rossmann P, Schallberger UH (2000). HAWIK-III Hamburg-Wechsler-Intelligenztest für Kinder [Wechsler Intelligence Scale for Children (WISC-III)-German Version].

[67] Moll K, Landerl K (2010). SLRT-II: Lese-und Rechtschreibtest.

[68] 68Blomert, L. & Vaessen, A. *Differentiaal Diagnostiek van Dyslexie: Cognitieve analyse van lezen en spellen [Dyslexia Differential Diagnosis: Cognitive analysis of reading and spelling]*. (Boom Test Publishers, 2009).

[69] Achenbach, T. M. & Edelbrock, C. S. *Manual for the Child Behavior Checklist and Revised Child Behavior Profile*. (Department of Psychiatry, University of Vermont, 1983)

[70] Abdullaev YG, Posner MI (1998). Event-related brain potential imaging of semantic encoding during processing single words. Neuroimage.

[71] Wilke M, Holland SK, Altaye M, Gaser C (2008). Template-O-Matic: A toolbox for creating customized pediatric templates. Neuroimage.

[72] Mazaika, P., Whitfield-Gabrieli, S., Reiss, A. & Glover, G. In *Organization of Human Brain Mapping International Conference*.

[73] Tzourio-Mazoyer N (2002). Automated anatomical labeling of activations in SPM using a macroscopic anatomical parcellation of the MNI MRI single-subject brain. Neuroimage.

[74] Pedhazur, E. J. *Multiple regression in behavioral research: explanation and prediction (2nd edition).* (Harcourt Brace Jovanovich, 1982).

[75] Cohen, J., Cohen, P., West, S. G. & Aiken, L. S. *Applied multiple regression/correlation analysis for the behavioral sciences.* (Lawrence Erlbaum Ass., 2003).

[76] Roth RM (2007). Event-related functional magnetic resonance imaging of response inhibition in obsessive-compulsive disorder. Biol. Psychiatry.

[77] Linkersdorfer J, Lonnemann J, Lindberg S, Hasselhorn M, Fiebach CJ (2012). Grey matter alterations co-localize with functional abnormalities in developmental dyslexia: An ALE meta-analysis. PLoS ONE.

[78] Richlan F, Kronbichler M, Wimmer H (2013). Structural abnormalities in the dyslexic brain: A meta-analysis of voxel-based morphometry studies. Hum. Brain. Mapp..

[79] Boros M (2016). Orthographic processing deficits in developmental dyslexia: Beyond the ventral visual stream. Neuroimage.

[80] Blomert L (2011). The neural signature of orthographic-phonological binding in successful and failing reading development. Neuroimage.

[81] Fraga Gonzalez G (2015). A randomized controlled trial on the beneficial effects of training letter-speech sound integration on reading fluency in children with dyslexia. PLoS ONE.

[82] Hoeft F (2006). Neural basis of dyslexia: A comparison between dyslexic and nondyslexic children equated for reading ability. J. Neurosci..

[83] Stevens WD, Kravitz DJ, Peng CS, Henry Tessler M, Martin A (2017). Privileged functional connectivity between the visual word form area and the language system. J. Neurosci..

[84] Vogel AC, Miezin F, Petersen SE, Schlaggar BL (2011). The putative visual word form area is functionally connected to the dorsal attention network. Cereb. Cortex.

[85] Karipidis II (2017). Neural initialization of audiovisual integration in prereaders at varying risk for developmental dyslexia. Hum. Brain Mapp..

[86] van de Walle de Ghelcke A, Rossion B, Schiltz C, Lochy A (2020). Developmental changes in neural letter-selectivity: A 1-year follow-up of beginning readers. Dev. Sci..

[87] Vogel AC, Miezin FM, Petersen SE, Schlaggar BL (2012). The putative visual word form area is functionally connected to the dorsal attention network. Cereb. Cortex.

[88] Chen C (2007). Sex determines the neurofunctional predictors of visual word learning. Neuropsychologia.

[89] Vidyasagar TR, Pammer K (2010). Dyslexia: A deficit in visuo-spatial attention, not in phonological processing. Trends Cogn. Sci..

[90] Grainger J, Dufau S, Ziegler JC (2016). A vision of reading. Trends Cogn. Sci..

[91] Galuschka K, Ise E, Krick K, Schulte-Korne G (2014). Effectiveness of treatment approaches for children and adolescents with reading disabilities: A meta-analysis of randomized controlled trials. PLoS ONE.

[92] Mehringer H (2020). (Swiss) GraphoLearn: An app-based tool to support beginning readers. Res. Pract. Technol. Enhanc. Learn..

[93] Franceschini S (2013). Action video games make dyslexic children read better. Curr. Biol..

[94] Franceschini S (2017). Action video games improve reading abilities and visual-to-auditory attentional shifting in English-speaking children with dyslexia. Sci. Rep..

[95] Breznitz Z (2013). Enhanced reading by training with imposed time constraint in typical and dyslexic adults. Nat. Commun..

[96] Hancock R, Richlan F, Hoeft F (2017). Possible roles for fronto-striatal circuits in reading disorder. Neurosci. Biobehav. Rev..

[97] Hickok G, Poeppel D (2007). The cortical organization of speech processing. Nat. Rev. Neurosci..

[98] Brown S, Ngan E, Liotti M (2008). A larynx area in the human motor cortex. Cereb. Cortex.

[99] Richlan F (2014). Functional neuroanatomy of developmental dyslexia: The role of orthographic depth. Front. Hum. Neurosci..

[100] Landerl K (2013). Predictors of developmental dyslexia in European orthographies with varying complexity. J. Child Psychol. Psychiatry.

[101] Seymour PH, Aro M, Erskine JM (2003). Foundation literacy acquisition in European orthographies. Br. J. Psychol..

[102] Hoeft F (2007). Functional and morphometric brain dissociation between dyslexia and reading ability. Proc. Natl. Acad. Sci. USA.

[103] Rorden C, Karnath HO, Bonilha L (2007). Improving lesion-symptom mapping. J. Cogn. Neurosci..

