# Supplementary material for: Visual word form processing deficits driven by severity of reading impairments in children with developmental dyslexia
Source: Sci Rep. 2020 Oct 30;10:18728. doi: 10.1038/s41598-020-75111-8 (PMC7603304; doi:10.1038/s41598-020-75111-8)
Supplement: Supplementary file 1 — Supplementary Information. [file 41598_2020_75111_MOESM1_ESM.docx]

**Visual word form processing deficits driven by severity of reading impairments in children with developmental dyslexia**

Brem S. ^1,2*^, Maurer U. ^1,3,4*^, Kronbichler M. ^5,6^, Schurz M. ^5^, Richlan F. ^5^, Blau V. ^7,8^, Reithler J. ^7,8^, van der Mark S. ^9^, Schulz E. ^10,11^, Bucher K. ^9^, Moll, K. ^11^, Landerl K. ^12,13^, Martin E. ^9^, Goebel R. ^7,8^, Schulte-Körne, G. ^11^, Blomert L. ^7,8#^, Wimmer H. ^5^, Brandeis, D. ^1,2,14^

^1^ Department of Child and Adolescent Psychiatry and Psychotherapy, Psychiatric Hospital, University of Zurich, Zurich, Switzerland

^2^ Neuroscience Center Zurich, University of Zurich and ETH Zurich, Zurich, Switzerland

^3^ Department of Psychology, The Chinese University of Hong Kong, Hong Kong, China

^4^ Brain and Mind Institute, The Chinese University of Hong Kong, Hong Kong, China

^5^ Centre for Cognitive Neuroscience and Department of Psychology, University of Salzburg, Salzburg, Austria

^6^ Neuroscience Institute, Christian Doppler Clinic, Paracelsus Medical University, Salzburg, Austria

^7^ Cognitive Neuroscience Department, Faculty of Psychology and Neuroscience, Maastricht University, Maastricht, the Netherlands

^8^ Maastricht Brain Imaging Center (M-BIC), Faculty of Psychology and Neuroscience, Maastricht University, Maastricht, the Netherlands

^9^ MR-Center, University Children's Hospital, University of Zürich, Zürich, Switzerland

^10^ Department of Neurology, Ludwig-Maximilians-Universität München, Munich, Germany

^11^ Department of Child and Adolescent Psychiatry, Psychosomatics, and Psychotherapy, University Hospital, Ludwig-Maximilians-University Munich, Munich, Germany

^12^ Department of Psychology, University of Salzburg, Salzburg, Austria

^13^ Institute of Psychology, University of Graz, Graz, Austria

^14^ Department of Child and Adolescent Psychiatry and Psychotherapy, Central Institute of Mental Health, Medical Faculty Mannheim, Heidelberg University

^*^ The first and second author contributed equally to this manuscript.

^#^ Our co-author Leo Blomert unfortunately passed away in November 2012, but his prior contributions to this project remain significant.

|  |
| --- |

**Corresponding author:**

Silvia Brem

Department of Child and Adolescent Psychiatry and Psychotherapy, Psychiatric Hospital, University of Zurich, Zurich, Switzerland

Neumuensterallee 9; CH-8032 Zurich; Switzerland

Email: [sbrem@kjpd.uzh.ch](mailto:urs.maurer@psychologie.uzh.ch)

Phone: ++41 44 499 2760

Running title: word processing deficits in dyslexia

Keywords: Dyslexia, reading, visual word form processing, development, children, fMRI, multicentre study

# Supplementary Material

*Supplementary analysis 1: Site-wise statistics*

Site-wise linear-mixed-model analysis using word reading fluency score as a covariate of interest and factors *ROI* (WFA, LFA) and *condition* (words, symbols):

Site-wise analyses yielded for the largest sample in Zürich (n=80) a significant interaction of *reading fluency* and *condition* (F(1,228)=7.63, p=0.0062) and a trend for *ROI x condition* (F(1,228)=3.01, p=0.0844). In addition, main effects of *ROI* (F(1,228)=48.62, p<0.0001) and *condition* (F(1,228)=16.31, p<0.0001) were also significant. For site Maastricht (n=35) trends for the interaction of *reading fluency* and *ROI* (F(1,97)=3.03, p<0.0850) and *reading fluency x ROI x condition* (F(1,97)=3.61, p<0.0604) were found. The data of the site Salzburg (n=25) only yielded an effect of *ROI* (F(1,69)=5.51, p<0.0218), which may be explained by the low sample size.

*Supplementary analyses 2: Group differences in print-sensitive vOT activation including the group of intermediate readers*

An additional LMM included the fixed factors *ROI* (WFA, LFW), *condition* (Words, Symbols) and reading group (dyslexia, intermediate readers, typical readers) and random factors *site* (SBG, ZRH, MAS) and subject as well as the covariate age. To account for possible differences between sites, we included site as a random factor in our LMM. Activation in the vOT ROIs corrected for *age* and *site* showed a trend for stronger activation to words than symbol strings (*condition*, F(1,406)=2.8, p<0.0950) and overall activation in the LFA was stronger than activation in the WFA (*ROI*, F(1,406)=15.55, p<0.0001). The word-sensitive activation tended to differ among the three groups (*group x condition x ROI*, F(2,406)=2.72, p=0.0669). The *group* main effect was not significant (*group*, F(2,406)=0.57, p=0.5674). Posthoc t-tests showed that only the typical readers had a more pronounced BOLD signal to words than symbol strings (print sensitive processing) in the WFA (t=3.59, p=0.0192) and less activation to symbol strings in the WFA than the LFA (t=-5.64 p=<0.001). Children with dyslexia showed a trend towards more pronounced activation in the LFA than WFA to words (t=-3.23, p=0.0594). The results with the three groups thus largely correspond to the main results with the core groups as detailed in the main text. Due to the considerable differences in groups sizes (intermediate group with only 12 subjects) we computed an additional analysis including the children with dyslexia (n=55) and by defining an “extended intermediate group” as children having a reading z-score (-1.25 < extended intermediate group ≤ -0.4; n=29) and a good readers group with reading fluency z-scores > -0.4 (>34.46 percentile, n=56). This additional model revealed again comparable results for the main effects as in previous analyses (*condition*, F(1,407)=4.79, p<0.0292; *ROI*, F(1,407)=32.92, p<0.0001; *group*, F(2,407)=0.33, p=0.7196) and approached a trend for interaction (*group x condition x ROI*, F(2,407)=2.31, p=0.1005). Posthoc t-tests showed that only the good readers had a more pronounced BOLD signal to words than symbol strings (print sensitive processing) in the WFA (t=3.55, p=0.0214), whereas for the extended intermediate (t=0.28, p=1) and dyslexia groups (t=0.76, p=0.9998) no condition effect in the WFA was found.

*Supplementary Figure 1: Words and Symbols vs baseline contrasts*


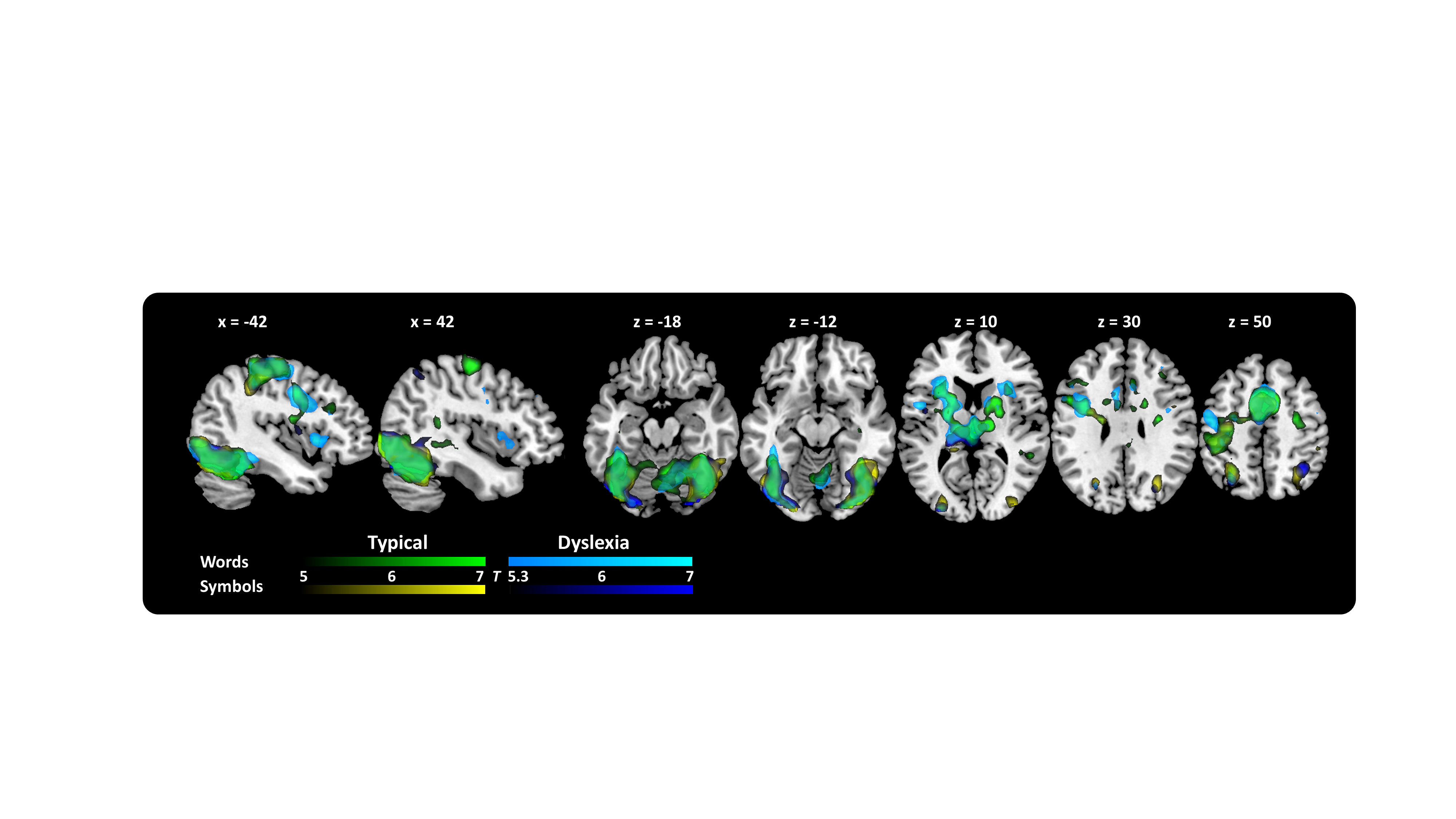


# *Two sagittal (MNI x =-42, x = 42) and five axial (MNI z=-18, z=-12, z=10, z=30, z=50) slices for the contrasts words > baseline (green: typical readers, light blue: dyslexia) and symbols > baseline (yellow: typical readers, dark blue: dyslexia). Activation threshold for all clusters and both groups illustrate at: p_(FWEp)_<0.05 (corresponding to a t> 5.06 for typical readers and t> 5.28 for dyslexia). The activation blobs are overlaid onto the ch2better.nii template using mricron (*[*https://www.nitrc.org/projects/mricron*](https://www.nitrc.org/projects/mricron)*) [*[*1*](#_ENREF_1)*].*

*Table S1: Results whole brain analyses, condition vs baseline*

| **Typical readers (n=73)** | | | | | | | |
| --- | --- | --- | --- | --- | --- | --- | --- |
| **Words vs. baseline** | | | | | | | |
| ***Hemisphere*** | ***Brain area*** | ***(MNI) x y z*** | ***k*** | ***Cluster p(FWEc)*** | ***Peak p(FWEp)*** | ***T*** | ***Z*** |
| L/R | medial frontal gyrus | -2 9 48 | 3258 | 0 | 0 | 13.06 | Inf |
| R/L | fusiform gyrus | 37 -51 -21 | 2672 | 0 | 0 | 12.32 | Inf |
| R | precentral gyrus | 40 -15 57 | 224 | 0 | 0 | 7.94 | 6.68 |
|  | undef. | 31 -9 30 | 18 | 0 | 0 | 7.59 | 6.46 |
| R | superior frontal gyrus | 19 42 24 | 9 | 0.001 | 0 | 7.17 | 6.19 |
| L | inferior frontal gyrus | -38 24 27 | 50 | 0 | 0 | 6.59 | 5.79 |
| R | middle/superior temporal gyrus | 43 -39 15 | 37 | 0 | 0 | 6.48 | 5.71 |
|  | undef. | 7 0 27 | 6 | 0.003 | 0.001 | 6.2 | 5.51 |
|  | undef. | 16 30 0 | 9 | 0.001 | 0.001 | 6.15 | 5.48 |
| R | middle frontal gyrus | 34 33 30 | 8 | 0.002 | 0.004 | 5.82 | 5.23 |
| R | hippocampus | 37 -21 -12 | 7 | 0.002 | 0.005 | 5.76 | 5.19 |
| L | fusiform gyrus | -38 -18 -21 | 5 | 0.004 | 0.008 | 5.66 | 5.12 |
|  | undef. | 52 15 -6 | 6 | 0.003 | 0.008 | 5.66 | 5.12 |
|  |  |  |  |  |  |  |  |
| **Symbols vs. baseline** | |  |  |  |  |  |  |
| R | inferior occipital gyrus | 37 -84 -9 | 1197 | 0 | 0 | 12.23 | Inf |
| L/R | medial frontal gyrus | -5 3 51 | 328 | 0 | 0 | 11.1 | Inf |
| L | middle occipital gyrus | -29 -90 -6 | 867 | 0 | 0 | 10.31 | Inf |
| L | inferior parietal lobule | -29 -54 45 | 244 | 0 | 0 | 9.12 | 7.38 |
|  | undef. | -17 -36 15 | 19 | 0 | 0 | 8.08 | 6.77 |
| L | pre/postcentral gyrus | -20 -9 33 | 608 | 0 | 0 | 7.93 | 6.68 |
| R | angular gyrus/precuneus | 28 -60 39 | 200 | 0 | 0 | 7.23 | 6.23 |
| L | cerebellum | -2 -78 -27 | 27 | 0 | 0 | 6.57 | 5.78 |
| R | middle frontal gyrus | 34 0 60 | 8 | 0.002 | 0.001 | 6.11 | 5.45 |
|  | undef. | -2 -27 12 | 21 | 0 | 0.003 | 5.9 | 5.3 |
|  | undef. | -17 15 24 | 7 | 0.003 | 0.003 | 5.87 | 5.28 |
| R | inferior parietal lobe | 49 -36 48 | 15 | 0 | 0.005 | 5.75 | 5.19 |
| R | cerebellum | 7 -63 -24 | 12 | 0.001 | 0.008 | 5.62 | 5.09 |
| R | precentral gyrus | 40 -15 54 | 5 | 0.005 | 0.012 | 5.49 | 4.99 |
|  | | | | | | | |
| **Dyslexia (n=55)** | |  |  |  |  |  |  |
| **Words vs. baseline** | |  |  |  |  |  |  |
| L | putamen | -20 9 3 | 1537 | 0 | 0 | 14.73 | Inf |
|  | medial frontal gyrus | -2 0 54 | 755 | 0 | 0 | 13.65 | Inf |
| R | fusiform gyrus/ cerebellum | 40 -54 -24 | 918 | 0 | 0 | 11.82 | Inf |
| L | cerebellum/ fusiform gyrus | -32 -51 -30 | 700 | 0 | 0 | 10.07 | 7.47 |
| R | putamen /inferior frontal gyrus | 22 12 0 | 309 | 0 | 0 | 8.89 | 6.9 |
| L | superior parietal Lobule | -29 -63 45 | 77 | 0 | 0 | 7.32 | 6.04 |
| L | postcentral gyrus | -53 -21 24 | 12 | 0 | 0 | 6.97 | 5.83 |
| R | inferior frontal gyrus | 64 6 18 | 13 | 0 | 0 | 6.84 | 5.75 |
| R | precentral gyrus | 34 -18 60 | 18 | 0 | 0.001 | 6.57 | 5.58 |
| L | inferior parietal lobule | -44 -36 45 | 10 | 0.001 | 0.002 | 6.29 | 5.4 |
| R | middle frontal gyrus | 37 39 33 | 10 | 0.001 | 0.002 | 6.28 | 5.39 |
| L | middle frontal gyrus | -32 42 24 | 9 | 0.001 | 0.003 | 6.16 | 5.31 |
| R | precentral gyrus | 49 -3 48 | 9 | 0.001 | 0.005 | 6 | 5.21 |
| R | precentral gyrus | 40 0 30 | 5 | 0.003 | 0.006 | 5.98 | 5.19 |
|  | undef. | -38 24 21 | 8 | 0.001 | 0.008 | 5.9 | 5.14 |
| R | superior temporal gyrus | 49 -27 0 | 8 | 0.001 | 0.011 | 5.8 | 5.07 |
|  |  |  |  |  |  |  |  |
| **Symbols vs. baseline** | |  |  |  |  |  |  |
| L | fusiform gyrus/ middle occipital gyrus | -29 -90 -6 | 756 | 0 | 0 | 12.38 | Inf |
| R | middle occipital gyrus/ fusiform gyrus | 37 -84 0 | 1102 | 0 | 0 | 11.08 | Inf |
|  | medial frontal gyrus/ cingulate gyrus | -8 6 48 | 339 | 0 | 0 | 9.67 | 7.28 |
| L | thalamus | -11 -24 9 | 268 | 0 | 0 | 9.54 | 7.22 |
| L | precentral gyrus | -38 -24 66 | 415 | 0 | 0 | 8.82 | 6.87 |
| L | precuneus | -26 -63 42 | 91 | 0 | 0 | 8.3 | 6.6 |
| R | putamen | 22 12 0 | 57 | 0 | 0 | 8.3 | 6.59 |
| R | inferior parietal lobule | 34 -51 45 | 137 | 0 | 0 | 7.75 | 6.29 |
| L | putamen | -23 6 0 | 40 | 0 | 0 | 7.37 | 6.07 |
| R | thalamus | 16 -30 0 | 14 | 0 | 0 | 6.99 | 5.84 |
|  | undef. | 25 -69 3 | 14 | 0 | 0 | 6.89 | 5.78 |
| L | precentral gyrus | -44 0 6 | 12 | 0 | 0.002 | 6.27 | 5.39 |
| R | caudate | 13 -6 15 | 5 | 0.003 | 0.023 | 5.54 | 4.89 |

*For all contrasts: significance level at whole-brain peak-level threshold p_(FWEp)_ < 0.05, k≥5 Labels of brain regions were determined using the XJView (AAL atlas); k: cluster size; R: right; L: left.*

*References*

1 Rorden, C., Karnath, H. O. & Bonilha, L. Improving lesion-symptom mapping. *J Cogn Neurosci* **19**, 1081-1088, doi:10.1162/jocn.2007.19.7.1081 (2007).
